# Association between Dietary Inflammatory Index Scores and Diabetes Sensorimotor Polyneuropathy in Patients with Type 2 Diabetes Mellitus: A Case-Control Study

**DOI:** 10.1155/2022/2661649

**Published:** 2022-04-21

**Authors:** Sara Asadi, Azadeh Aminianfar, Fahimeh Shiva, Sasan Asadi, Habib Yarizadeh, Mostafa Qorbani, Khadijeh Mirzaei

**Affiliations:** ^1^Department of Community Nutrition, School of Nutritional Sciences and Dietetics, Tehran University of Medical Sciences, Tehran, Iran; ^2^Students' Scientific Research Center, Tehran University of Medical Sciences, Tehran, Iran; ^3^Research Center for Biochemistry and Nutrition, Metabolic Disease, Kashan University of Medical Sciences, Kashan, Iran; ^4^Department of Community Nutrition, School of Nutritional Sciences and Dietetics, Isfahan University of Medical Sciences, Isfahan, Iran; ^5^Department of Community Medicine, School of Medicine, Kurdistan University of Medical Sciences, Sanandaj, Kurdistan Province, Iran; ^6^Non-communicable Diseases Research Center, Alborz University of Medical Sciences, Karaj, Iran; ^7^Chronic Diseases Research Center, Endocrinology and Metabolism Population Sciences Institute, Tehran University of Medical Sciences, Tehran, Iran

## Abstract

**Background:**

Diabetes sensorimotor polyneuropathy (DSPN) is a common complication of diabetes. Diet has been previously related to DSPN. However, no studies have investigated the relationship between the inflammatory potential of the whole diet and DSPN. So, we aimed to examine the association between dietary inflammatory index (DII) and DSPN in Iranian adults.

**Methods:**

A total of 185 subjects with DSPN and 185 sex- and age-matched controls were selected in this case-control study. A 168-item validated food frequency questionnaire (FFQ) was used to assay dietary intakes. DII was calculated based on the developed formula. The Toronto clinical neuropathy score was applied to define DSPN. Binary logistic regression was used to estimate the odds ratios (ORs) and 95% confidence intervals (95% CIs) of DII in relation to DSPN.

**Results:**

Mean values of age and BMI for all the participants were reported as 50.79 and 28.60, respectively. Also, the median (IQR) of DII for all the participants was estimated as −0.091 (−0.93, 1.07). Our findings suggest that participants in the highest quartile of the DII had higher odds of DSPN (OR = 1.76; 95% CI: 1.03, 3.36) (*p*-trend = 0.01) compared to subjects in the lowest quartile of DII scores after adjustment for age and sex. Additionally, a strong association was observed after adjusting for energy intake, physical activity, education, smoking status, economic status, marital status, job, BMI, and WC in model 2 (OR = 2.23, 95% CI = 1.13–4.39) (*p*-trend = 0.0048).

**Conclusion:**

Higher DII score was associated with an increased risk of DSPN. Therefore, it is possible that a diet rich in anti-inflammatory nutrients and foods could improve and prevent DSPN.

## 1. Introduction

Diabetes mellitus (DM) is a major metabolic disorder. It was estimated that 422 million people worldwide have been diagnosed with diabetes, which is the largest global epidemic of the 21st century [1]. There are many complications associated with diabetes, including diabetes sensorimotor polyneuropathy (DSPN). The DSPN is typically associated with a higher risk of falling, pain, depression, anxiety, and decreased quality of life [2, 3]. Moreover, DSPN is a major contributor to neuropathic ulcers and infection, which can cause lower limb amputation [4]. There has been evidence that chronic systemic inflammation contributes to noncommunicable diseases, including cancer, cardiovascular disease, depression, and diabetes [5, 6]. In fact, inflammation, oxidative stress, induced by chronic hyperglycemia, and mitochondrial dysfunction are the three most important risk factors in the pathogenesis of DSPN [7].

Research shows that DSPN can be caused not only by hyperglycemia but also by other factors including overweight and obesity [8], sedentary lifestyles and smoking [9], and unhealthy diets [10]. Therefore, regulating blood glucose through diet, exercise, or medication can play an important role in preventing and management of diabetes and associated complications such as DSPN [11]. The linkage between diet and inflammation and subsequent increase in the risk of developing type 2 diabetes mellitus (T2DM) have been shown in previous studies. For example, Schulze [12] showed that a diet high in sugar-sweetened soft drinks, refined grains, and processed meat but low in wine, coffee, and cruciferous vegetables can increase inflammation and the risk of developing diabetes. A diet rich in red meat and saturated fat is associated with the activation of proinflammatory factors [13]. However, a diet high in whole grains, fruits, and vegetables can inhibit the production of proinflammatory cytokines including tumor necrosis factor-*α* (TNF-*α*) and interleukin-1 (IL-1) [14]. The dietary inflammatory index (DII) was designed to represent the total proinflammation of a whole diet [15]. Also, some studies have demonstrated an association between DII score and inflammatory biomarkers [16, 17]. Based on the results of a cross-sectional study, a proinflammatory diet can raise the risk of DM among adult Mexicans [18]. Among a cohort of Iranian adults, a cross-sectional study showed that DII had a positive association with postload glucose (2h-PG) [19]. Additionally, a case-control study showed that patients with an anti-inflammatory diet were at a lower risk of prediabetes compared to patients with the proinflammatory diet [20]. Furthermore, the metabolic syndrome and a variety of chronic diseases have been associated with DII [21–23]. To the best of our knowledge, no study has addressed an association between the DII and DSPN. We aimed to examine the association between DII and DSPN in a case-control study.

## 2. Methods

### 2.1. Study Design and Subjects

This case-control study was carried out in the Diabetes Research Center, Endocrinology and Metabolism Clinical Sciences Institute, Kermanshah, Iran. The present study included 185 cases with noninsulin-dependent diabetes mellitus (NIDDM), aged 30–60 years with body mass index (BMI) between 25 and 39.9 kg/m^2^ and diagnosed with DSPN caused by diabetes and 185 age (±10), sex-matched controls with NIDDM, and lack of DSPN. Also, this study is a revised version of the preprint, which has been previously published in Research Square [24]. Patients were excluded based on the following criteria: (1) those diagnosed with neuropathy due to other diseases, (2) patients with a history of diseases such as cancer, and liver, kidney, autoimmune, inflammatory, thyroid, and nervous and cardiovascular diseases, and (3) pregnant or lactating individuals. Patients were also excluded if they had a particular diet during the last two months. Also, we excluded subjects from the analysis if they had unexplained total energy intake (<800 Kcal/day or >4200 Kcal/day). The study protocol was approved by the Ethics Committee of Tehran University of Medical Science, TUMS.VCR.REC. 1399.269. All the participants were required to sign written informed consent.

The following equation was used to calculate the sample size of the study:

(1) $$\begin{array}{c} N=\frac{{\left(Z_{1-\left(\alpha /2\right)}+Z_{1-\beta }\right)}^{2}p_{1}\left(1-p_{1}\right)p_{2}\left(1-p_{2}\right)}{{\left(p_{2}-p_{1}\right)}^{2}}, \end{array}$$

where *p*_1_ is the proportion exposed to DII in the control group and *p*_2_ is the proportion exposed to DII in the case group. Assuming *α* = 0.05 and *β* = 0.2 and the prevalence of diabetes in the least anti-inflammatory diet group and the most anti-inflammatory diet group to be equal to 6.4 and 22.8, respectively [18], we needed 185 participants in each group.

### 2.2. Dietary Assessment and Dietary Inflammatory Index Calculation

Dietary intakes were assessed using a valid and reliable semiquantitative Food Frequency Questionnaire (FFQ) (168 food items, with standard serving sizes as commonly consumed by Iranians) [25]. One trained expert completed all the FFQ questionnaires. All the participants were asked about their average dietary intake on a daily, weekly, and monthly basis during the last year. Daily nutrients and energy intakes of foods and beverages were analyzed by Nutritionist software version 4 (First Data Bank, San Bruno, CA), modified for Iranian foods.

The DII was estimated using FFQ and Shivappa et al. [15] In this regard, we included 29 parameters to calculate DII, which were energy, carbohydrate, fat, protein, fiber, cholesterol, monounsaturated fatty acids (MUFAs), polyunsaturated fatty acids (PUFAs), saturated fats (SFAs), cobalamin, pyridoxine, folic acid, niacin, riboflavin, thiamin, vitamin A, C, D, E, *β*-carotene, zinc, selenium, magnesium, iron, caffeine, garlic, onion, pepper, and green/black tea. First, we calculated the *Z* score for all the 29 parameters by the ratio of the standard global mean from the quantity of food parameters consumed by each participant to the global standard deviation. We obtained the global mean and SD from Shivappa et al. [15] This value was converted to the percentile score. Then, the result of this value was multiplied by the effect score for all of the food parameters gained from Shivappa et al. [15] Eventually, the DII score from all the foods was summed to compute an overall DII score for each subject.

### 2.3. Toronto Clinical Neuropathy Score

The Toronto clinical neuropathy score (TCNS) was employed to diagnose and compute the severity of neuropathy. It has been proved that TCNS is a valid and reliable questionnaire in Iran and other countries [26, 27]. The TCNS contains three sections: the first section included symptoms scores that were calculated based on the absence or presence of foot pain, numbness, tingling, and weakness in the feet, similar upper-limb symptoms, and ataxia (six points). Symptom scores were rated as absence = 0 or presence = 1. The second section included sensory test scores calculated based on the absence or presence of pinprick, temperature, light touch, vibration, and position sense, which were performed at the first toe (five points). Scores were rated as absence = 0 or presence = 1. Finally, the third section included reflexes scores (knee reflexes and ankle reflexes) rated as normal = 0, reduced = 1, or absence = 2 (eight points). The TCNS scores range from a minimum of 0 (without neuropathy) to a maximum of 19 points. Based on the result of TCNS, subjects were graded into classes of no neuropathy: 0–5; mild neuropathy: 6–8; moderate neuropathy: 9–11; and severe neuropathy >12. Clinical examinations were conducted by a neurologist and the results were recorded on a special form.

### 2.4. Assessment of Other Variables

We evaluated the economic status using a validated and reliable questionnaire for the Iranian population [28]. This questionnaire includes items about ownership of a vehicle or house, possession of modern furniture, number of family members, and travels in or outside the country during the past year. Based on this questionnaire, the economic score ranged from 0 to 9, in which a higher score represented a good economic status. In this study, participants were categorized into the group of low economic status 0–3, mild economic status 3–6, and high economic status 7–9. A digital scale was used to determine the weight to the nearest 50 g. Also, the height was measured to the nearest 0.5 cm. Participants were asked not to wear shoes. The BMI was computed through weight (kg) divided by height squared (m^2^). A nonelastic tape with an accuracy of 0.5 cm was used to measure waist circumference (WC) at a point midway between the iliac crest and lower rib margin. The short form of the International Physical Activity Questionnaire (IPAQ) was employed to estimate physical activity during the last week. The IPAQ is a validated self-reported seven-item measure of physical activity during the last week [29]. The anthropometric measurements were performed by a dietitian trained in anthropometry to reduce individual error.

We took fasted venous blood samples from all the participants. We separated the blood sample by centrifugation (at 3000 rpm for 10 min at 4°C) to acquire serum. Then, they were stored at −80°C until biochemical analyses were performed. An auto-analyzer instrument (ERBA), using commercial kits (Pars Azmoon, Iran), was used to measure fasting blood sugar (FBS) and blood sugar after 2 hours (Bs2hp). Also, we used a high-performance liquid chromatography (HPLC) (Advance Scientific Instrument, Germany) to estimate glycated hemoglobin (HbA1c).

### 2.5. Statistical Analysis

Data were analyzed using SPSS 16 for Windows (SPSS Inc., Chicago, IL). First, quartile cut-off points for the DII score were defined. All the patients were classified based on the following cut-offs: *q*1 <−0.93, *q*2 (−0.93) − (−0.09), *q*3 (−0.09) −(1.07), and *q*4 <1.07. To compare the means and distribution of continuous and categorical variables, an independent sample *t*-test and analysis of the Chi-square test were run, respectively. Differences across quartiles of DII were compared using the one-way ANOVA procedure and Chi-square test. Food and nutrient intakes were adjusted for age, sex, and energy, except for dietary energy intake, which was only adjusted for age and sex using ANCOVA by quartiles of the DII. Binary logistic regression was employed to investigate associations between the DII and DSPN. Three regression models were used in our analysis. In model 1, it was adjusted for age, sex, and energy intake. In model 2, we adjusted further for physical activity, education, economic status, and smoking. Moreover, in model 3, it was adjusted for BMI to perceive the relationship regardless of obesity. Also, binary logistic regression was run to calculate P trend in all the models by considering the DII score as a continuous variable. We defined the first quartile of DII as the reference category and odds ratios and 95% CIs for the other quartiles were estimated. *p* values <0.05 were set as statistically significant.

## 3. Results

In the present study, the mean values of age and BMI for all the participants were 50.79 and 28.60, respectively. Also, the median (IQR) of DII for all the participants was −0.091 (−0.93, 1.07).

The general characteristics of participants are provided in Table 1. There were significant differences between both groups in terms of BMI (*p*=0.002) and HbA1C (*p*=0.03). Cases and controls were not significantly different in terms of mean age, BS2Hp, job, marriage, gender, physical activity, economic status, and education. Also, compared to the control group, the cases reported more smoking rate (*p*=0.02). Additionally, there were significant differences in terms of marriage (*p* < 0.001) and smoking (*p*=0.04) across the quartiles of the DII score.

Selected nutrients and food group intakes of participants are listed in Table 2. There was a significant difference between cases and controls in terms of PUFA, MUFA, Mg, thiamine, folate, dietary fiber, whole grains, red meat, fish, and vegetables. Moreover, there were significant differences in terms of red meats (*p*=0.04) and fruits (*p*=0.02) across the quartile of the DII score. Participants assigned in the lowest category of DII score were characterized by higher intake of energy (*p* < 0.001), carbohydrates (*p* < 0.001), protein (*p* < 0.001), PUFA (*p* < 0.001), Fe (*p* < 0.001), Mg (*p* < 0.001), Zn (*p* < 0.001), niacin (*p* < 0.001), vitamin A (*p* < 0.001), folate (*p* < 0.001), thiamin (*p* < 0.001), vitamin C (*p*=0.03), vitamin E (*p* < 0.001), *β*-carotene (*p* < 0.001), fish (*p* < 0.001), vegetables (*p* < 0.001), dietary fiber (*p* < 0.001), white meats (*p*=0.01), refine grains (*p*=0.04), and dairy products (*p* < 0.001).

Table 3 lists the multivariable-adjusted ORs and 95% CIs for the association between the DII score and the odd of DSPN. There was a significant trend across the quartiles of DII for the crude model (OR = 1.76, 95% CI = 1.03–3.36) (*p*-trend = 0.01). Compared with the first quartile and after adjustment for age and sex in model 1 (OR = 1.76; 95% CI: 1.03, 3.36) (*p*-trend = 0.01), a significant association was observed. Moreover, a strong relationship was observed after adjusting for a wide range of confounding including energy intake, physical activity, education, smoking status, economic status, marital status, job, BMI, and WC in the model 2 (OR = 2.23, 95% CI = 1.13–4.39) (*p* trend = 0.0048).

## 4. Discussion

Reportedly, no study has yet explored the relationship between the DII score and the risk of DSPN. Our results indicated that subjects in the highest quartile of DII score (the most proinflammatory diet) had higher odds of DSPN (independent of other risk factors) than subjects in the lowest quartile of the DII score (the most anti-inflammatory diet).

Our findings are consistent with the hypothesis that diet plays an important role in the prevention or control of DSPN through the regulation of chronic inflammation [30, 31]. In an animal study, inflammation has been considered a major risk factor in the pathogenies of DSPN [32, 33]. The inflammatory potential of a diet can play an important role in the development and pathogenies of diseases including T2DM [18] and cancers [34]. However, there has been no study on an association between DII and DSPN. The range of DII (defined by Shivappa et al. [15]) in our study (ranging from −0.93 to +1.07) was similar to that reported in an Italian population [35]. In the present investigation, a positive relationship was observed between DII and the odds of having DSPN. In line with our findings, the results of a case-control study conducted on women showed that a diet with high inflammation potential could result in higher inflammatory markers and increase the development of type 2 diabetes [12]. Another cross-sectional study investigated the relationship between DII and T2DM in a Mexican population. The results showed that patients with a higher DII score had about three times greater odds of diabetes compared to subjects with the lower DII score [18]. In addition, investigating the association between the DII and prediabetes revealed that patients with a higher DII score were at increased odds of prediabetes compared to subjects who had a more anti-inflammatory diet [19]. Additionally, the anti-inflammatory diet was associated with higher fasting and Bs2hp levels and greater insulin resistance, as reported by Van Woudenbergh et al. [36]. In contrast, our findings showed no relationship between DII and type 2 diabetes. This can be due to methodological differences between this study and earlier research, which employed as follows: different designs, different types and numbers of dietary components used in the DII calculation, and different covariate availabilities for multivariate analyses [19, 21].

The etiology of DSPN is complex and multifactorial. The mechanisms between the anti-inflammatory diet and DSPN could be explained by improving hyperglycemia that has been related to the inflammatory process. Chronic hyperglycemia results in the activation of several pathways including the elevation of protein kinase C, polyol pathway, glucose autoxidation, and hexosamine flux. Therefore, hyperglycemia through these pathways activates proinflammatory cytokines such as interleukin 1, interleukin 6, and finally, nuclear factor kappa-light-chain-enhancer of activated B cells (NF-KB), which play a central role in inflammation [37–39]. Furthermore, various flavonoids, alkaloids, and steroids of anti-inflammatory diet may reduce pain in patients with DSPN by the inhibition of prostaglandin synthesis, a peripheral mechanism of pain inhibition [40].

As the first strength of this study, an association between DII and DSPN was examined. In addition, it was adjusted for various covariates that could change the results. Moreover, new cases of DSPN were enrolled in order to reduce the bias from changed dietary habits in participants.

There were also some limitations in this research. First, our findings cannot be used to infer causal relationships because of the observational method of study. Second, the FFQ was used for assessing food intake, which carried recall bias where misclassification remains a possibility. Third, data on some food parameters were not available to calculate the DII score such as eugenol, ginger, n-3 and n-6 fatty acids, saffron, trans fat, turmeric, thyme/oregano, rosemary, and flavonoids, which may have influenced our results.

## 5. Conclusion

In conclusion, our findings suggest that a higher DII score could be associated with an increased odd ratio of DSPN. Therefore, this study could provide some evidence to support the recommendation of low consumption of proinflammatory foods and a high intake of foods with anti-inflammatory potential. However, further prospective and trial studies in different populations are required to clarify the role and effect of DII on the development of DSPN.

## Figures and Tables

**Table 1:** General characteristics of study participants.

	Cases (*n* = 185)	Controls (*n* = 185)	*p*	Dietary inflammatory index quartiles				
				1 (*n* = 91) <−0.93	2 (*n* = 90) (−0.93) − (−0.09)	3 (*n* = 92) (−0.09) − (1.07)	4 (*n* = 91) 1.07<	*p*
Age (years)	50.4 ± 8.2	50.7 ± 7.3	0.9^a^	50.9 ± 7.6	49.3 ± 8.4	52.5 ± 6.7	50.8 ± 8.1	0.05^b^
BMI (kg/m^2^)	28.9 ± 2.3	28.2 ± 2.3	0.002^a^	28.7 ± 2.3	28.3 ± 2.7	28.6 ± 2.2	28.7 ± 2.2	0.7^b^
Waist circumstance (cm)	99.5 ± 14.9	101.6 ± 8.6	0.1^a^	101.1 ± 9.6	100 ± 9.2	99.1 ± 8.6	101.3 ± 18.8	0.6^b^
FBS (mg/dl)	161.7 ± 58.2	165.3 ± 53.4	0.5^a^	159.4 ± 56.4	167.2 ± 57.6	165.5 ± 55.5	162.6 ± 55.1	0.8^b^
BS2hp (mg/dl)	242.9 ± 86.4	226 ± 80.7	0.5^a^	229.3 ± 79.3	229.2 ± 92.1	235 ± 77.8	246 ± 85.3	0.4^b^
HbA1c	8.2 ± 1.5	7.9 ± 1.4	0.03^a^	8.2 ± 1.5	7.9 ± 1.5	8.02 ± 1.4	8.08 ± 1.4	0.6^b^
Physical activity (MET-min/week)^d^			0.3^c^					0.4^c^
Low intensity (%)	61.6	67.6		61.5	60.9	64.8	71.1	
Moderate (%)	30.3	27		33	30.4	25.3	25.6	
High intensity (%)	8.1	5.4		5.5	8.7	9.9	3.3	
Females (%)	63.8	63.8	0.5^c^	64.8	58.7	65.9	58	0.7^c^
Married (%)	99.5	100	0.66^c^	25	25.5	27	23	<0.001^c^
Education (%)			0.5^c^					0.9^c^
Literate (%)	94.1	94.6		94.5	95.6	93.5	93.4	
Illiterate (%)	5.9	5.4		5.5	4.4	6.5	6.6	
Current smoker (%)	10.3	4.3	0.02^c^	4.4	13	3.3	8.9	0.04^c^
Economic status^e^			0.5^c^					
Low (%)	15.1	17.3		15.4	19.6	14.3	16.7	0.5^c^
Middle (%)	83.3	79.5		82.4	79.3	81.3	81.1	
High (%)	1.6	3.2		2.2	1.1	4.4	2.2	
Job			0.2^c^					0.2^c^
Working (%)	43.8	48.6		42.9	55.4	45.1	43.3	
Housewife	56.2	51.4		57.1	44.6	54.9	56.7	

The results are described as mean ± standard deviation or (%). BS2hP, blood sugar after 2 hrs; BMI, body mass index; FBS, fasting blood sugar; HbA1C, glycated hemoglobin A1c; MET, metabolic equivalent of task. ^a^Independent sample *t*-test. ^b^ANOVA test. ^c^Chi‐square test. ^d^Low intensity: lower than 600 MET‐min/week; moderate: 600–3,000 MET‐min/week; high intensity: more than 3,000 MET‐min/week. ^e^Economic status: low: 0–3; middle: 4–6; high:7–9.

**Table 2:** Dietary intakes of patients.

	Cases (*n* = 185)	Controls (*n* = 185)	*p* ^*∗*^	Dietary inflammatory index quartiles				*p* ^*∗*^
				1 (*n* = 91) <−0.93	2 (*n* = 90) (−0.93) − (−0.09)	3 (*n* = 92) (−0.09) − (1.07)	4 (*n* = 91) 1.07<	
	Mean ± SD	Mean ± SD		Mean ± SD	Mean ± SD	Mean ± SD	Mean ± SD	
Energy (kcal/d)	2506 ± 461	2487 ± 331	0.6	2692 ± 424	2549 ± 342	2486 ± 334	2255 ± 357	<0.001
Nutrients								
Carbohydrates (g/d)	407.2 ± 75.9	394.2 ± 74.6	0.08	443.6 ± 61.9	406 ± 48.2	404 ± 61.1	354.9 ± 72.5	<0.001
Proteins (g/d)	121 ± 245.4	117 ± 160.7	0.8	115.1 ± 20.3	106 ± 23.8	99.9 ± 19.2	96.4 ± 45.1	<0.001
Total fats (g/d)	79.2 ± 38.3	81.5 ± 40.4	0.5	82.3 ± 26.4	77.3 ± 28.2	77.5 ± 28.4	76.7 ± 28.3	0.5
Cholesterol (mg/d)	227.4 ± 53	224.7 ± 65.1	0.5	237.5 ± 47	235.1 ± 70.5	224.5 ± 50.9	211.7 ± 59.3	0.02
SFA (g/d)	23.2 ± 8.7	21.2 ± 16.4	0.1	22 ± 4.55	20.4 ± 3.67	21.4 ± 4.01	21.6 ± 5.55	0.1
MUFA (g/d)	26.5 ± 4.2	23.8 ± 4.3	<0.001	27.1 ± 4.28	24.9 ± 3.61	24.5 ± 3.61	24.2 ± 5.24	<0.001
PUFA (g/d)	18.7 ± 3.4	16.8 ± 2.9	<0.001	19.8 ± 2.88	17.4 ± 2.78	17.1 ± 2.33	16.4 ± 3.27	<0.001
Fe (mg/d)	33.9 ± 117.5	30.6 ± 85.09	0.8	27.9 ± 4.14	25.2 ± 3.03	24.1 ± 3.03	21.9 ± 4.78	<0.001
Mg (mg/d)	522.5 ± 114.2	580 ± 132.6	<0.001	643.3 ± 128	576.5 ± 82.8	515.4 ± 74.6	470.6 ± 127	<0.001
Zn (mg/d)	17.9 ± 52.1	17.2 ± 32.1	0.8	16.5 ± 2.50	15 ± 1.97	13.8 ± 1.74	12.6 ± 2.91	0.001
Se (mg/d)	201.1 ± 191.1	202.6 ± 191.6	0.9	210.9 ± 41.1	195.6 ± 33.1	176.1 ± 30.1	228 ± 378	0.3
Thiamine (mg/d)	3 ± 0.70	2.8 ± 0.54	0.01	3.2 ± 0.50	3.01 ± 0.42	2.9 ± 0.43	2.6 ± 0.51	<0.001
Riboflavin (mg/d)	2.6 ± 1.98	2.6 ± 1.96	0.8	2.7 ± 0.56	2.4 ± 0.32	2.4 ± 0.29	2.8 ± 3.9	0.4
Niacin (mg/d)	32.3 ± 6.5	31.5 ± 9.4	0.3	36.4 ± 5.34	33.6 ± 9.16	31.6 ± 7.22	27.7 ± 5.89	<0.001
Vitamin B6 (mg/d)	5.4 ± 43.5	5.4 ± 43.3	0.9	2.3 ± 0.33	2.1 ± 0.43	1.9 ± 0.37	15 ± 86.9	0.1
*β*-Carotene (mcg/d)	3112 ± 948.5	2959 ± 2307.7	0.3	4051 ± 3067	2911 ± 840	2916 ± 753	2438 ± 575	<0.001
Vitamin A (RE/d)	619.9 ± 412.6	605.6 ± 461.2	0.7	758 ± 409	552 ± 141	518 ± 115	547 ± 514	<0.001
Vitamin C (mg/d)	99.9 ± 250.7	143 ± 312.8	0.1	132 ± 138	96.1 ± 26.2	91.8 ± 27.8	92 ± 170	0.05
Vitamin E (mg/d)	13.6 ± 3.89	13.9 ± 3.13	0.3	16 ± 3.64	14.3 ± 3.19	13.4 ± 2.44	11.5 ± 2.81	<0.001
Vitamin D (mcg/d)	1.9 ± 2.75	2.1 ± 1.7	0.3	2.7 ± 0.66	1.8 ± 0.68	1.8 ± 0.81	1.8 ± 2.23	0.6
Vitamin B12 (mcg/d)	10.5 ± 81.4	8.7 ± 62.9	0.8	4.6 ± 1.79	4 ± 1.28	3.8 ± 0.80	4.8 ± 6.17	0.1
Folate (mcg/d)	765 ± 161.3	715 ± 115.1	<0.001	841.1 ± 114	743.1 ± 102	744.3 ± 107	643.3 ± 119	<0.001
Dietary fiber (g/d)	82.7 ± 27.6	68.8 ± 17.7	<0.001	85 ± 22.2	74.3 ± 21.8	78.8 ± 22.1	69.3 ± 23.8	0.001
Food groups								
Refined grains (g/d)	414 ± 149.5	398 ± 131.8	0.2	443 ± 127	381 ± 143	414 ± 125	378 ± 152	0.02
Whole grains (g/d)	227.6 ± 121.7	255.2 ± 122.5	0.03	267 ± 123.2	251 ± 131.9	216 ± 102.7	233 ± 130.8	0.03
White meats (g/d)	41.5 ± 15.3	48.7 ± 61.8	0.1	47.2 ± 15.4	54.9 ± 71.5	45.4 ± 51.5	32.8 ± 12.24	0.01
Red meats	9.3 ± 4.9	8.2 ± 3.9	0.01	9.3 ± 4.76	7.9 ± 3.55	9.4 ± 4.89	8.2 ± 4.33	0.04
Processed meats (g/d)	3.8 ± 3.04	3.7 ± 2.51	0.5	5.9 ± 2.86	6.2 ± 3.40	6.3 ± 2.30	6 ± 2.38	0.9
Fish (g/d)	4.1 ± 3.22	4.9 ± 4.01	0.03	5 ± 3.36	4.1 ± 3.55	4.5 ± 4.15	4.3 ± 3.56	0.4
Fruits (g/d)	173.2 ± 61.2	181.4 ± 44.5	0.1	177 ± 48.1	188 ± 47.3	173 ± 55.3	165 ± 51.9	0.02
Vegetables (g/d)	386.7 ± 119.3	417.1 ± 118.9	0.01	483 ± 135.2	394 ± 93.4	407 ± 94	327 ± 97.1	<0.001
Dairy products (g/d)	340.1 ± 158.9	346.7 ± 112.9	0.6	392 ± 126.2	346 ± 124.2	340 ± 112.6	288 ± 156.5	<0.001

^*∗*^All values were adjusted for age, sex, and energy, except for dietary energy intake, which was adjusted only for age and sex using ANCOVA.

**Table 3:** Odds ratios (ORs) and 95% confidence intervals (95% CIs) of DSPN according to quartiles of dietary inflammatory index.

	Dietary inflammatory index quartiles				*p* trend^*∗*^
	1 (*n* = 91) <−0.93	2 (*n* = 90) (−0.93) − (−0.09)	3 (*n* = 92) (−0.09) − (1.07)	4 (*n* = 91) <1.07	
	OR	OR (95% CI)	OR (95% CI)	OR (95% CI)	

Crude	1.00	0.79 (0.39–1.28)	1.27 (0.71–2.27)	1.76 (1.03–3.36)	0.01
Model 1	1.00	0.79 (0.39–1.27)	1.27 (0.71–2.27)	1.76 (1.03–3.36)	0.01
Model 2	1.00	0.69 (0.37–1.31)	1.21 (0.64–2.26)	2.24 (1.13–4.39)	0.008

^*∗*^Binary logistic regression was used to obtain OR and 95% CI. The overall trend of OR across increasing quartile was examined by considering the median score in each category as a continuous variable. Model 1: adjusted for age (continuous) and sex (male/female). Model 2: further adjustments were made for energy intake (kJ/d or kcal/d), physical activity (low intensity, moderate, or high intensity), education (literate or illiterate), smoking status (yes or no), economic status (low, middle, or high), marital status (married or single), job (working or housewife), BMI (continuous), and WC (continuous).

## Data Availability

The dataset used in this paper is available from the corresponding author upon request.

## References

[1] World Health Organization (2019). *Classification of Diabetes Mellitus*.

[2] Callaghan B. C. (2020). Diabetic neuropathy: the future is promising. Reply to Uusitupa M, Niskanen L, Laitinen T [letter] and Coppini DV. *Diabetologia*.

[3] Girach A. (2019). Quality of life in painful peripheral neuropathies: a systematic review. *Pain Research and Management*.

[4] Gylfadottir S. S., Weeracharoenkul D., Andersen S. T., Niruthisard S., Suwanwalaikorn S., Jensen T. S. (2019). Painful and non-painful diabetic polyneuropathy: clinical characteristics and diagnostic issues. *Journal of diabetes investigation*.

[5] Fung T. T., McCullough M. L., Newby P. (2005). Diet-quality scores and plasma concentrations of markers of inflammation and endothelial dysfunction. *The American Journal of Clinical Nutrition*.

[6] Rahman M. M. (2022). Citrus limon L.(lemon) seed extract shows neuro-modulatory activity in an in vivo thiopental-sodium sleep model by reducing the sleep onset and enhancing the sleep duration. *Journal of Integrative Neuroscience*.

[7] Halim M., Halim A. (2019). The effects of inflammation, aging and oxidative stress on the pathogenesis of diabetes mellitus (type 2 diabetes). *Diabetes and Metabolic Syndrome: Clinical Research and Reviews*.

[8] Visser M. (1999). Elevated C-reactive protein levels in overweight and obese adults. *JAMA*.

[9] Petersen A. M. W., Pedersen B. K. (2005). The anti-inflammatory effect of exercise. *Journal of Applied Physiology*.

[10] Calder P. C. (2011). Dietary factors and low-grade inflammation in relation to overweight and obesity. *British Journal of Nutrition*.

[11] Zilliox L. A., Russell J. W. (2019). Physical activity and dietary interventions in diabetic neuropathy: a systemic review. *Clinical Autonomic Research*.

[12] Schulze M. B., Hoffmann K., Manson J. E. (2005). Dietary pattern, inflammation, and incidence of type 2 diabetes in women. *The American Journal of Clinical Nutrition*.

[13] Carvalho C. A., Silva A. A. M., Assunção M. C. F. (2019). The dietary inflammatory index and insulin resistance or metabolic syndrome in young adults. *Nutrition*.

[14] Arabzadegan N., Daneshzad E., Fatahi S., Moosavian S. P., Surkan P. J., Azadbakht L. (2019). Effects of dietary whole grain, fruit, and vegetables on weight and inflammatory biomarkers in overweight and obese women. *Eating and Weight Disorders-Studies on Anorexia*.

[15] Shivappa N., Steck S. E., Hurley T. G., Hussey J. R., Hébert J. R. (2014). Designing and developing a literature-derived, population-based dietary inflammatory index. *Public Health Nutrition*.

[16] Shivappa N., Hébert J. R., Rietzschel E. R. (2015). Associations between dietary inflammatory index and inflammatory markers in the Asklepios Study. *British Journal of Nutrition*.

[17] Shivappa N., Steck S. E., Hurley T. G. (2014). A population-based dietary inflammatory index predicts levels of C-reactive protein in the Seasonal Variation of Blood Cholesterol Study (SEASONS). *Public Health Nutrition*.

[18] Denova-Gutiérrez E., Muñoz-Aguirre P., Shivappa N. (2018). Dietary inflammatory index and type 2 diabetes mellitus in adults: the diabetes mellitus survey of Mexico City. *Nutrients*.

[19] Moslehi N., Ehsani B., Mirmiran P. (2016). Inflammatory properties of diet and glucose-insulin homeostasis in a cohort of Iranian adults. *Nutrients*.

[20] Vahid F., Shivappa N., Karamati M., Naeini A. J., Hebert J. R., Davoodi S. H. (2017). Association between dietary inflammatory index (DII) and risk of prediabetes: a case-control study. *Applied Physiology Nutrition and Metabolism*.

[21] Alkerwi A. A., Shivappa N., Crichton G., Hébert J. R. (2014). No significant independent relationships with cardiometabolic biomarkers were detected in the observation of cardiovascular risk factors in luxembourg study population. *Nutrition Research*.

[22] Shivappa N., Zucchetto A., Montella M. (2015). Inflammatory potential of diet and risk of colorectal cancer: a case-control study from Italy. *British Journal of Nutrition*.

[23] Neufcourt L. (2016). Prospective association between the dietary inflammatory index and cardiovascular diseases in the supplémentation en vitamines et minéraux antioxydants (SU.VI.MAX) cohort. *Journal of American Heart Association*.

[24] Asadi S., Aminianfar A., Asadi S., Yarizadeh H., Qorbani M., Mirzeai K. (2020). *F. Shiva Higher Dietary Inflammatory Index Scores Are Associated with Increased Odds of Diabetic Sensorimotor Polyneuropathy in Patients with Type 2 Diabetes Mellitus: A Case-Control Study*.

[25] Hosseini Esfahani F., Asghari G., Mirmiran P., Azizi F. (2010). Reproducibility and relative validity of food group intake in a food frequency questionnaire developed for the tehran lipid and glucose study. *Journal of Epidemiology*.

[26] Bostani A., Homayounfar H. (2006). The relationship between ncs findings and toronto clinical scoring system of neuropathy in diabetic polyneuropathy. *Journal of Kermanshah University of Medical Sciences*.

[27] Bril V., Perkins B. A. (2002). Validation of the Toronto clinical scoring system for diabetic polyneuropathy. *Diabetes Care*.

[28] Garmarodi G. R., Moradi A. (2010). Socio-economic status in Iran: a study of measurement index. *Payesh (Health Monitor)*.

[29] Aadahl M., Jorgensen T. (2003). Validation of a new self-report instrument for measuring physical activity. *Medicine and Science in Sports and Exercise*.

[30] Chrysohoou C., Panagiotakos D. B., Pitsavos C., Das U. N., Stefanadis C. (2004). Adherence to the Mediterranean diet attenuates inflammation and coagulation process in healthy adults. *Journal of the American College of Cardiology*.

[31] Cameron N., Cotter M. (2008). Pro-inflammatory mechanisms in diabetic neuropathy: focus on the nuclear factor kappa B pathway. *Current Drug Targets*.

[32] Kellogg A. P., Wiggin T. D., Larkin D. D., Hayes J. M., Stevens M. J., Pop-Busui R. (2007). Protective effects of cyclooxygenase-2 gene inactivation against peripheral nerve dysfunction and intraepidermal nerve fiber loss in experimental diabetes. *Diabetes*.

[33] Wang Y., Schmeichel A. M., Iida H., Schmelzer J. D., Low P. A. (2006). Enhanced inflammatory response via activation of NF-*κ*B in acute experimental diabetic neuropathy subjected to ischemia-reperfusion injury. *Journal of the neurological sciences*.

[34] Huang W.-Q., Mo X.-F., Ye Y.-B. (2017). A higher Dietary Inflammatory Index score is associated with a higher risk of breast cancer among Chinese women: a case-control study. *British Journal of Nutrition*.

[35] Shivappa N., Bosetti C., Zucchetto A., Serraino D., La Vecchia C., Hébert J. R. (2015). Dietary inflammatory index and risk of pancreatic cancer in an Italian case-control study. *British Journal of Nutrition*.

[36] van Woudenbergh G. J., Theofylaktopoulou D., Kuijsten A. (2013). Adapted dietary inflammatory index and its association with a summary score for low-grade inflammation and markers of glucose metabolism: the cohort study on diabetes and atherosclerosis maastricht (CODAM) and the hoorn study. *The American journal of clinical nutrition*.

[37] Pop-Busui R., Ang L., Holmes C., Gallagher K., Feldman E. L. (2016). Inflammation as a therapeutic target for diabetic neuropathies. *Current Diabetes Reports*.

[38] Edwards J. L., Vincent A. M., Cheng H. T., Feldman E. L. (2008). Diabetic neuropathy: mechanisms to management. *Pharmacology and Therapeutics*.

[39] Harati Y. (1987). Diabetic peripheral neuropathies. *Annals of Internal Medicine*.

[40] Rahman M., Rajib A. H., Majumder S. (2021). Pre-clinical investigation of analgesic anti-diarrheal and CNS depressant effect of pterocarpus indicus in Swiss albino mice. *Jordan Journal of Pharmaceutical Sciences*.

